# Malaria diagnosis in a malaria non-endemic high-resource country: high variation of diagnostic strategy in clinical laboratories in the Netherlands

**DOI:** 10.1186/s12936-021-03889-7

**Published:** 2021-10-19

**Authors:** Marrit B. Boonstra, Rob Koelewijn, Eric A. T. Brienen, Welmoed Silvis, Foekje F. Stelma, Theo G. Mank, Bert Mulder, Lisette van Lieshout, Jaap J. van Hellemond

**Affiliations:** 1 Department of Medical Microbiology and Infectious Diseases, Erasmus MC University Medical Center, Rotterdam, The Netherlands; 2 Department of Parasitology, Leiden University Medical Centre, Leiden, The Netherlands; 3 Laboratory for Medical Microbiology and Public Health (LabMicTA), Hengelo, The Netherlands; 4 Department of Medical Microbiology, Radboudumc, Nijmegen, The Netherlands; 5 Regional Laboratory for Medical Microbiology and Public Health, Haarlem, The Netherlands; 6 Department of Medical Microbiology, Canisius-Wilhelmina Hospital, Nijmegen, The Netherlands

**Keywords:** Malaria, *Plasmodium*, Diagnosis, Microscopy, Methods, Quality control

## Abstract

**Background:**

Microscopic examination of thick and thin blood films is the gold standard in current guidelines for the diagnosis of malaria, but guidelines do not uniformly agree on which combination of other methods should be used and when.

**Methods:**

Three questionnaires were sent between March 2018 and September 2019 to laboratories subscribing to the external quality assessment scheme for the diagnosis of blood and intestinal parasites of the Dutch Foundation for Quality Assessment in Medical Laboratories in order to investigate how much variation in the laboratory diagnosis of malaria between different clinical laboratories is present in the Netherlands.

**Results:**

The questionnaires were partially or fully completed by 67 of 77 (87%) laboratories. Only 9 laboratories reported 10 or more malaria positive patients per year. Most laboratories use a different diagnostic strategy, within office versus outside office hours depending on the screening assay result. Within office hours, 62.5% (35/56) of the responding laboratories perform an immunochromatographic test (ICT) in combination with microscopic examination of thick and thin blood films without additional examinations, such as Quantitative Buffy Coat and/or rtPCR analysis. Outside office hours 85.7% (48/56) of laboratories use an ICT as single screening assay and positive results are immediately confirmed by thick and thin blood films without additional examinations (89.6%, 43/48). In case of a negative ICT result outside office hours, 70.8% (34/48) of the laboratories perform microscopic examination of the thick film the next morning and 22.9% (11/48) confirm the negative ICT result immediately. Furthermore, substantial differences were found in the microscopic examinations of thick and thin blood films; the staining, theoretical sensitivity of the thick film and determination of parasitaemia.

**Conclusions:**

This study demonstrated a remarkably high variation between laboratories in both their diagnostic strategy as well as their methods for microscopic examination for the diagnosis of malaria in a clinical setting, despite existing national and international guidelines. While the impact of these variations on the accuracy of the diagnosis of malaria is yet unknown, these findings should stimulate clinical laboratories to critically review their own diagnostic strategy.

**Supplementary Information:**

The online version contains supplementary material available at 10.1186/s12936-021-03889-7.

## Background

The Netherlands is a malaria non-endemic, high resource country in which on average only 150–300 imported malaria cases per year are reported by over 70 hospital organizations, that each often comprise multiple locations for emergency care [1–3]. Several laboratories consist of sub laboratories situated at multiple different locations that serve multiple hospitals. Furthermore, these malaria cases are most likely not evenly distributed across the country, which will lead to a limited experience of many physicians and laboratories in diagnosing malaria. Since the clinical presentation of malaria is mostly non-specific, the laboratory diagnosis of malaria is pivotal for adequate and timely treatment. Therefore, the use of high quality and reliable diagnostic methods is crucial.

Microscopic examination of thick and thin blood films is the gold standard in current guidelines and the most widely used method for the diagnosis of malaria and identification of the *Plasmodium* species. However, microscopic examination may be combined with other diagnostic methods, such as an immunochromatographic test (ICT), Quantitative Buffy Coat (QBC) examination and nucleic acid detection methods (e.g. loop-mediated isothermal amplification (LAMP) or real-time polymerase chain reaction (rtPCR)). Guidelines for the laboratory diagnosis of malaria do not uniformly agree on how to integrate the ICT in the laboratory diagnostic strategy for malaria and are not clear on which of the above mentioned methods should be combined within and outside office hours. Therefore, it can be expected that substantial variations exist in the diagnostic strategies for malaria diagnosis between different clinical laboratories, a phenomenon which has also been noticed in two small surveys on malaria diagnostic practices in U.S. laboratories performed in 2010 and 2017 [4, 5]. However, the extent of these variations and its impact on the reliability and sensitivity of the diagnostic methods is unknown.

To investigate how much variation is present in the laboratory diagnosis of malaria in symptomatic patients between different clinical laboratories in the Netherlands, the following research questions were formulated: (i) what is the variation in diagnostic strategies (i.e. the combination of different methods) for malaria in laboratories in the Netherlands and (ii) to what extent do the laboratories differ in the microscopic examination of thick and thin blood films, the key method for the laboratory diagnosis. Therefore, a survey was conducted on the laboratory diagnostic strategy and microscopic examination details of thick and thin blood films to diagnose malaria in clinical laboratories in the Netherlands. In addition, the guidelines for malaria diagnosis of the most important authorities on microscopic examination of thick and thin blood films, were compared.

## Methods

### Questionnaire

Three anonymous questionnaires on the laboratory diagnostic strategy and details on the microscopic examination of thick and thin blood films to diagnose malaria were conducted between March 2018 and September 2019 as part of the regular external quality assessment scheme (EQAS) for blood and intestinal parasite diagnostics of the Dutch Foundation for Quality Assessment in Medical Laboratories (SKML). The questionnaires were in Dutch (see Additional file 1 for Dutch and Dutch to English translated questionnaires) and sent to all laboratories who subscribed to this scheme. Filling in the questionnaires is not an obligatory part of the scheme, but participants are always stimulated to report their answers to occasional additional theoretical questions. Laboratories reporting only EQAS results for intestinal parasites, were excluded. Participation to the EQAS of the SKML is not mandatory for Dutch laboratories, however in practice the vast majority of Dutch laboratories do participate.

### Data management and analysis

All answers to the questionnaires were automatically entered in Excel (Microsoft Office 2016) and subsequently reviewed and analysed. Several questions consisted of multiple choice options as well as a free-text option, meaning that the participating laboratories could enter additional textual comments to further explain their answer. If both options were filled in, the free-text answer overruled the multiple-choice answer in case of inconsistencies. In those cases where the answers to a multiple choice question were incongruent with other answers or with the provided additional textual comments, the correct interpretation was decided by a group of three authors. In case a free-text answer was really not clear, it was not included in the analysis.

### Diagnostic strategy questions

Diagnostic strategy is defined by the combination of different methods used for the diagnosis of malaria in symptomatic patients.

A screening assay is defined by an easy to use test method, such as an ICT or LAMP assay, that provides a fast presumptive diagnosis result and needs to be followed up by either a more sensitive test or a test that provides further information on *Plasmodium* species and/or parasitaemia.

### Microscopic examination questions

Laboratories were asked to indicate the extensiveness of microscopic examination of thick and thin blood films in either duration of examination in minutes or amount of microscopic fields examined, by ticking a box with a range of minutes or microscopic fields. Mean numbers of the reported range were used for the calculation of the theoretical limit of detection of the thick and thin blood film. If the exact number was given in the free-text option, this number was used for the calculation. Duration of examination in minutes for both thick and thin blood film was converted in number of microscopic fields by applying the assumption that the average laboratory staff is semi-experienced and therefore the conversion formula of 4 s per microscopic field was used [6].

For the calculation of the theoretical limit of detection of the thick film examination given in ‘total viewed volume’ and ‘number of trophozoites per microlitre (µL)’ several assumptions were made: (i) a Field Number (FN) of 18 (a diameter of 18 mm of the image area seen through the ocular) of the microscopes was assigned to laboratories using an ocular with 10× magnification [7] and a FN of 16 was assigned to the laboratories using an ocular with 12.5× magnification; (ii) the thick film is a perfect circle on which the blood is equally distributed; (iii) all red blood cells (RBCs) and parasites will remain attached to the slide after staining, although in practice some parasites will be lost during processing; and (iv) each microscopic field examined is a unique field, although in practice these fields can be overlapping.

For the calculation of the limit of detection of the thin film examination and determination of parasitaemia, the following assumptions were made: 1 µL of blood contains 5,000,000 RBCs and one microscopic field to contain 250 RBCs per microscopic field if a total magnification of 1000× was used [8]. For other magnifications, a proportional amount of erythrocytes per microscopic field was used. In addition it was assumed that each microscopic field examined through the microscope is a uniquely examined microscopic field.

## Results

### Surveyed laboratories and frequency of laboratory examinations for malaria

Of the 77 SKML blood parasitology EQAS subscribing laboratories 67 (87.0%) partially or fully completed the questionnaires. Table 1 shows the frequency of laboratory examinations for malaria and the reported number of malaria positive patients per year in the surveyed laboratories. Half of the laboratories (29/58) examine less than 50 requests per year and almost two third of the laboratories (36/55) diagnose less than 6 malaria patients per year (Table 1). Only 9 laboratories reported 10 or more malaria positive patients per year. If more malaria laboratory requests per technician per laboratory per year (by dividing the amount of laboratory requests per laboratory by the number of technicians performing malaria examinations per laboratory) was associated with a better overall performance score in the malaria EQAS from 2013 to 2020, was analysed using one-way analysis of variance and linear regression analysis. Although a trend was found, the correlation between the number of malaria requests per technician per laboratory per year and the overall performance score in the malaria EQAS was not statistically significant.

**Table 1:**
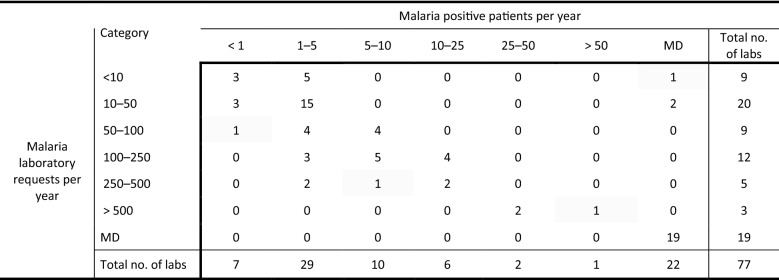
Characteristics of the 77 surveyed laboratories

MD: missing data; no. of labs: number of laboratories

### Laboratory diagnostic strategy

**Fig. 1:**
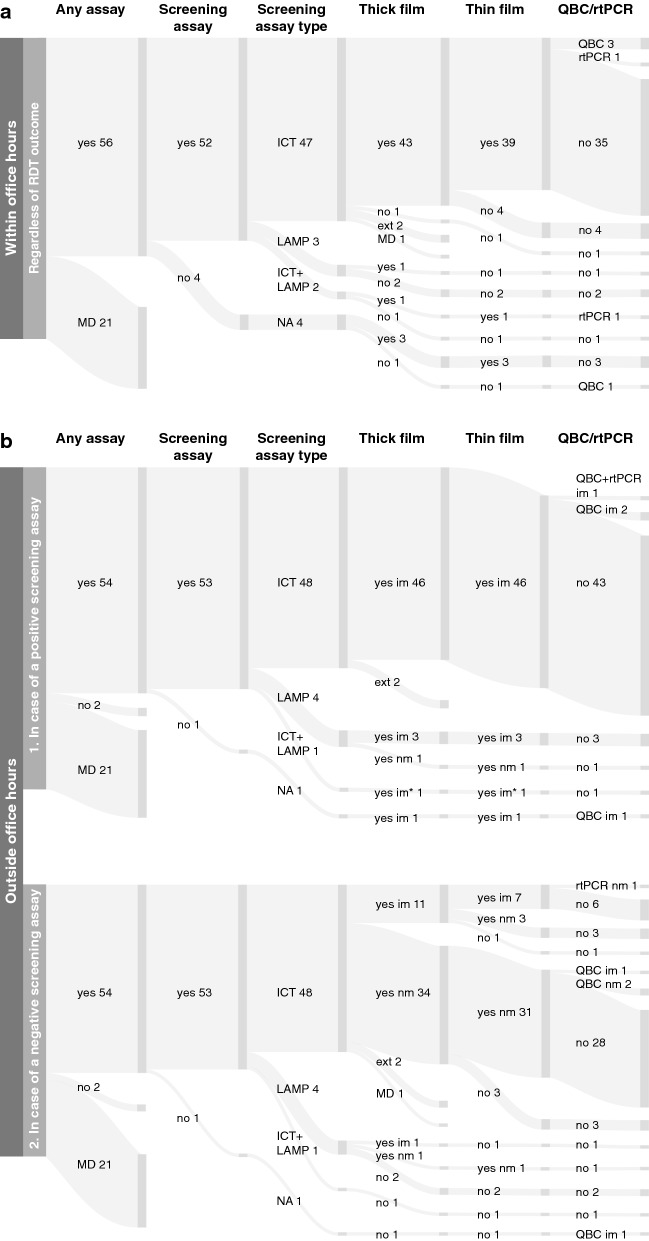
Sankey diagram showing the diagnostic strategy for methods to diagnose malaria within and outside office hours. The diagnostic strategy for the laboratory diagnosis of malaria within office hours (panel **A**) and outside office hours (panel **B**) are shown. Since outside office hours many laboratories use a different strategy depending on the result of the screening assay, panel B is split in two graphs showing the workflow in case of positive (panel B1) or negative test result of the screening assay (panel B2). MD: missing data; NA: not applicable; Ext: externally performed; LAMP: loop mediated isothermal amplification; ICT: immunochromatographic test; rtPCR: real-time polymerase chain reaction; QBC: Quantitative Buffy Coat; im: immediately; nm: next morning; *if histidine-rich protein 2 (HRP-2) antigen is positive in ICT

The surveyed laboratories perform different diagnostic strategies, depending on the time of day at which the examination has to be performed; within or outside office hours. Fifty-six (56/77, 72.7%) laboratories reported sufficient information on this subject. Figure 1 shows the diagnostic strategy for malaria examinations in the surveyed laboratories in three different settings: (A) within office hours; (B1) outside office hours in case of a positive test result of the screening assay (ICT and/or LAMP); (B2) outside office hours in case of a negative test result of the screening assay (ICT and/or LAMP).

#### Diagnostic strategy within office hours

More than half of the responding laboratories (35/56, 62.5%) perform a diagnostic strategy in which an ICT is used in combination with microscopic examination of thick and thin blood films without additional examinations, such as QBC and/or rtPCR analysis. Within office hours, 49 laboratories perform a commercial ICT: BinaxNOW^tm^ (n = 36); CareStart^tm^ (n = 1); OptiMAL-IT (n = 3); Palutop® + 4 OPTIMA (n = 1); SD BIOLINE Malaria Ag P.f/Pan (n = 3); Other (n = 1); Missing data (MD) (n = 4). Laboratories that perform LAMP analysis (n = 5), either without (n = 3) or in combination with ICT (n = 2), vary in performing additional microscopic examination of thick and thin blood films and rtPCR analysis, as some perform thick and thin blood film analysis in combination with rtPCR (1/5), some perform only thick blood film analysis (1/5) and some do not perform microscopic blood film analysis (3/5). Additional examinations (QBC and/or rtPCR) are only performed by a small number (6/56, 10.7%) of laboratories.

#### Diagnostic strategy outside office hours

Outside office hours, again 49 laboratories perform an ICT. The following commercial tests were used by laboratories outside office hours: BinaxNOW^tm^ (n = 36); OptiMAL-IT (n = 3); Palutop® + 4 OPTIMA (n = 1); SD BIOLINE Malaria Ag P.f/Pan (n = 4); Other (n = 1); MD (n = 4). Although the total number of laboratories performing an ICT are similar within or outside office hours, the set of laboratories are slightly different as 3 laboratories reported only performing an ICT within office hours and 3 laboratories reported only performing an ICT outside office hours.

Outside office hours and in case of a positive test result of the screening assay, laboratories are most agreeing on the diagnostic strategy: forty-three (43/56, 76.8%) laboratories use an ICT as the single screening assay outside office hours, perform microscopic examination of thick and thin blood films to confirm the positive screening assay result and do not perform additional rtPCR and/or QBC examinations. Similar to the diagnostic strategy used within office hours, variations exist in the diagnostic strategy in laboratories performing a LAMP assay.

Outside office hours and in case of a negative test result of the screening assay, the diagnostic strategy used is most diverse. In case of a negative ICT result outside office hours, the majority of laboratories do not directly perform microscopic examination of the thick film, but perform this analysis the next morning (34/48, 70.8%) and a minority of the laboratories confirms the negative ICT result immediately (11/48, 22.9%) by microscopic examination of the thick film. It is unknown whether three laboratories (3/48, 6.3%) confirm a negative ICT with microscopic examination immediately or the next morning (n = 2, externally performed; n = 1, missing data). In addition, even more variation can be seen in whether or not and when additional microscopic examination of the thin film is performed in case of a negative test result of the screening assay.

Altogether these results demonstrate that although microscopic examination of thick and thin blood films is the backbone of the laboratory diagnosis of malaria, substantial differences exist between different laboratories in which methods are performed and when, especially outside office hours in case of a negative test result of the screening assay.

### Staining of thick and thin blood films

Fifty (50/77, 64.9%) laboratories reported information about the staining method of thick and thin blood films. Forty-one (41/50, 82.0%) laboratories reported to use a Giemsa staining method as the only staining method for the examination of all thick and/or thin blood films. By far the most reported pH level used in Giemsa staining was 7.2 (range 7.0–7.2). Three (3/50, 6.0%) laboratories reported to use a Diff-Quik (modified Wright-Giemsa) staining only, one (1/50, 2.0%) laboratory uses both Diff-Quik and Giemsa staining and two (2/50, 4.0%) laboratories use Diff-Quik staining and additional Giemsa staining in case of doubt. Three (3/50, 6.0%) laboratories reported to use a May-Grünwald-Giemsa stain only with pH levels of 6.8, 7.0 and 8.0.

**Fig. 2:**
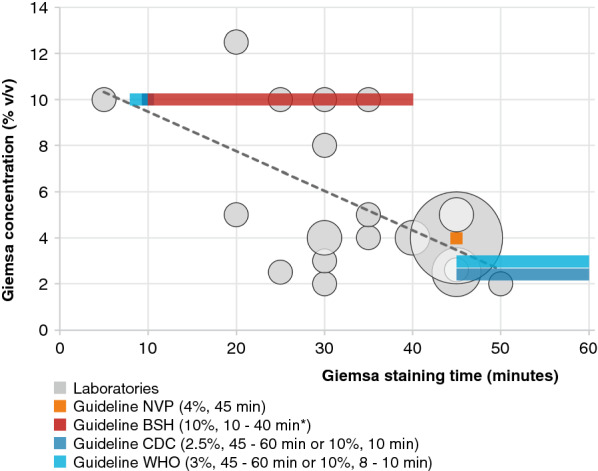
Giemsa concentration and staining time of thick and thin blood films. The size of the spheres from small to large represent 1, 2, 4 and 14 laboratories. The colored bars represent the different indicated guidelines

Figure 2 shows the substantial variation in the used concentration of Giemsa stain and the staining time of thick and thin blood films among the laboratories. Thirty-eight (38/42, 90.5%) laboratories using Giemsa staining method for all thick and/or thin blood films reported information about the concentration and staining time. The Giemsa stain concentration (v/v) ranged from 2 to 12.5% (mean 4.7%; median 4%) and the duration of Giemsa staining ranged from 5 to 50 min (mean 38 min; median 45 min). The trend line in Fig. 2 shows an inversed correlation between the used Giemsa concentration and staining time: the lower the Giemsa concentration, the longer the staining time. If a higher Giemsa concentration and a longer staining time correlated with the staining of thicker thick films was investigated, but due to missing data, the number of laboratories for which sufficient information was available to answer this question was too small. The combination of Giemsa stain concentration and staining time most used by the laboratories (14/38, 36.8%) is 4% (v/v) and 45 min. This combination is in accordance with the guideline of the Dutch Society for Parasitology (NVP) [9]. Guidelines on the Giemsa staining method for the diagnosis of malaria from the British Society for Haematology (BSH) [10], Center for Disease Control and Prevention (CDC) [11] and the World Health Organization (WHO) [8] differ substantially from each other as well and are also indicated in Fig. 2.

### Theoretical limit of detection of the thick and thin blood films

The theoretical limit of detection of the thick film could be calculated if sufficient information was reported on the following four criteria: (i) volume of blood used; (ii) diameter of thick film; (iii) total magnification used for microscopic examination; (iv) either duration of microscopic examination in minutes or amount of microscopic fields examined. Of all participants 21 (27.3%) laboratories reported this information (Table 2). The amount of µL blood used to prepare thick films varied from 6 to 15 µL (mean 8 µL; median 6 µL) and the reported diameter of the thick film varied from 6 to 23.25 mm (mean 14 mm; median 13 mm). Both NVP and WHO guidelines recommend to use 6 µL of blood for the preparation of the thick film with a diameter of 12 mm, however the guidelines differ in the recommendation of the minimum amount of microscopic fields that must be examined before a thick film can be declared as negative, respectively 200 versus 100 microscopic fields [8, 9, 12]. The theoretical limit of detection of the BSH and CDC guidelines could not be determined since the volume of the thick film and/or diameter are not specified in these guidelines [10, 11]. The theoretical limit of detection of the thin film could be calculated if sufficient information was given on: (i) total magnification used for microscopic examinations; (ii) either duration of microscopic examination in minutes or amount of microscopic fields examined. Laboratories that reported to refrain from examining the thin film if the thick film showed no parasites, were excluded from this calculation, since the thin film is then not a part of the procedure to detect parasites and thus does not add to the sensitivity component.

**Table 2:** Theoretical sensitivity of thick and thin blood films of surveyed laboratories (n = 50, MD = 27)

Guideline/laboratory	Total magnification (ocular × objective)	Thick film					Thin film		
		Volume of thick film	Diameter of thick film	Number of fields examined	Theoretical total examined volume	Theoretical limit of detection (based on 2 trophozoites)	Theoretical fields examined	Theoretical total examined volume	Theoretical part of volume examined compared to thick film
Guideline	*	µL	mm	Number	µL	Trophozoites /µL	Number	µL	%
NVP [9, 12]	1000	6	12	200	0.3	7.41	ND		
BSH [10]	1000^	Several drops	–	200			ND		
CDC [11]	1000^	Small drop	17.9**	100–300			–		
WHO [8]	1000	6	12	100	0.1	14.29	ND		
Range									
Minimum	500	6	6	75	0.09	1.2	40	0.002	0.8%
Maximum	1250	15	23.25*	413	1.65	21.9	600	0.056	10.3%
Mean	NA	8	14	207	0.47	7.4	190	0.010	3.4%
Median	NA	6	13	200	0.31	6.5	175	0.009	2.9%
Laboratory									
A	1000	6	20	188	0.09	21.9	188	0.009	10.3%
B	1000	–	–				263	0.013	
C	500	6	15	175	0.60	3.3	175	0.018	2.9%
D	1000	6	6	200	1.08	1.9	ND		
E	1000	–	–				338	0.017	
F	625	–	–				113	0.009	
G	500	–	–				188	0.019	
H	1000	–	–				188	0.009	
I	1000	6	13	225	0.26	7.7	ND		
J	1000	–	10				75	0.004	
K	1000	–	–				275	0.014	
L	1250	6	13	338	0.31	6.5	74	0.003	1.0%
M	1000	6	15	200	0.17	11.6	200	0.010	5.8%
N	1000	6	13	188	0.22	9.3	ND		
O	1000	–	–				263	0.013	
P	500	6	15	338	1.17	1.7	563	0.056	4.8%
Q	500	6	13	225	1.04	1.9	–		
R	500	–	–				125	0.006	
S	625	–	–				413	0.009	
T	1000	6	12	200	0.27	7.4	ND		
U	1000	–	–				40	0.002	
V	1000	6	11	200	0.32	6.2	ND		
W	1000	10	23.25	200	0.12	16.7	75	0.004	3.1%
X	1000	6	12	200	0.27	7.4	ND		
Y	1000	–	–				113	0.006	
Z	1000	10	9	413	1.65	1.2	413	0.021	1.3%
AA	1000	–	–				75	0.004	
AB	1000	6	–				49	0.002	
AC	1000	–	20				263	0.013	
AD	1000	–	–				113	0.006	
AE	1000	–	–				263	0.013	
AF	1000	6	12	200	0.27	7.4	–		
AG	1000	–	15				49	0.002	
AH	1000	–	–				263	0.013	
AI	1000	15	14	135	0.33	6.0	135	0.007	2.0%
AJ	1000	–	–				338	0.017	
AK	1000	–	–				175	0.009	
AL	1000	–	–				74	0.004	
AM	1000	–	–				200	0.010	
AN	1000	6	12	113	0.15	13.2	ND		
AO	1000	6	10	200	0.39	5.1	200	0.010	2.6%
AP	1000	6	–				75	0.004	
AQ	1000	6	–				600	0.030	
AR	1000	–	–				75	0.004	
AS	625	15	16	75	0.45	4.4	ND		
AT	1000	–	–				188	0.009	
AU	1000	15	–				74	0.004	
AV	1000	6	10	225	0.44	4.6	74	0.004	0.8%
AW	1000	12	15	113	0.19	10.3	113	0.006	2.9%
AX	1000	–	–				113	0.006	

–: missing data; .: no calculation possible due to missing data; ND: not determined for the detection of parasites; NA: not applicable

*The size of 1 euro coin

**The size of a US dollar dime

^1000× magnification and FN 18 of the ocular is assumed, based on the use of a 100× objective

Table 2 shows the theoretical limit of detection of the examination of thick and thin blood films (see Additional file 2 for more detailed information on the calculations). For 21 laboratories the total examined blood volume in the thick film could be calculated, which ranged from 0.09 to 1.65 µL. Assuming that at least two trophozoites need to be recorded for a positive result, a theoretical detection limit was calculated of 22 to 1.2 trophozoites per µL, respectively. This result shows that the theoretical sensitivity of thick film analysis differs by at least a factor 20 among clinical laboratories in the Netherlands.

For 40 laboratories the total examined volume in the thin film could be calculated, which ranged from 0.002 µL to 0.056 µL resulting in a theoretical detection limit of 1000 to 36 infected erythrocytes per µL, respectively. For 10 laboratories the theoretical total blood volume examined in both their thick and thin film analysis could be calculated, whereupon the theoretical proportion of volume examined in the thin film compared to the thick film was calculated. This proportion ranged from 0.8 to 10.3% and confirms the higher theoretical sensitivity of thick film examination compared to thin film examination.

### Determination of parasitaemia

**Fig. 3:**
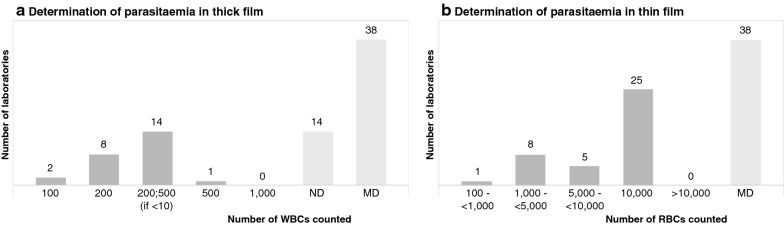
Determination of parasitaemia. **A** Shows the amount of WBCs counted in the thick films by the surveyed laboratories for the determination of parasitaemia. The guidelines count the following amount of WBCs: NVP (200 WBCs or 500 WBCs if < 10 parasites are counted); BSH (MD); CDC 1000 WBCs or 500 parasites, whichever comes first); WHO (200 WBCs or 500 WBCs if < 100 parasites are counted). **B** Shows the amount of RBCs in the thin films counted by the laboratories for the determination of parasitaemia. The guidelines count the following amount of RBCs: NVP (10,000 RBCs); BSH (1000 RBCs); CDC (500 RBCs, 2000 if parasitaemia is < 1%); WHO (5000 RBCs). MD: missing data; NP: not performed; WBCs: white blood cells; RBCs: red blood cells

In case of an infection with *Plasmodium falciparum* or *Plasmodium knowlesi*, the parasitaemia has to be determined, as this is a parameter to determine the disease severity. Figure 3 shows (A) the amount of white blood cells (WBCs) counted in thick films and (B) the amount of red blood cells counted in thin films for the determination of parasitaemia. The recommended number of cells to be counted by the guidelines of the NVP [12], BSH [10], CDC [11] and WHO [8], varies substantially and are also indicated in Fig. 3. Thirty-nine laboratories reported sufficient information on this subject. In these surveyed laboratories, the range of red blood cells (RBCs) counted in the thin film varied from 100 to 10,000 (mean 7656; median 10,000) and the range of WBCs counted in the thick film varied from 100 to 500 (see Additional file 3). The majority of laboratories (25/39, 64.1%) stop counting parasites if 10,000 RBCs are counted in the thin film, which is in accordance with the Dutch NVP guideline. Fourteen laboratories (14/39, 35.9%) report counting 200 WBCs in the thick film, and continue to count 500 in case less than 10 parasites are counted in the first 200 WBCs, which is in accordance with the method recommended by the NVP. Fourteen other laboratories never determine parasitaemia in the thick film. These results demonstrate that the accuracy by which the parasitaemia is determined differs substantially especially in thin film examination, as the number of counted red blood cells differs by at least a factor 100 among clinical laboratories in the Netherlands.

## Discussion

In this study, large variations are found between the surveyed laboratories in the diagnostic strategies for malaria examinations in symptomatic patients in a non-endemic setting. Most of the laboratories use different diagnostic strategies within office hours compared to outside office hours. For example within office hours most laboratories perform microscopic examination of thick and thin blood films immediately regardless of the outcome of additional examinations such as an ICT. A single laboratory, however, indicated that, within office hours, it does not perform further examinations in addition to ICT, which is not in accordance with national and internal guidelines. Outside office hours most laboratories use a screening assay (ICT or LAMP) after which microscopic examination of thick and thin blood films is performed the next morning in case of a negative screening assay result. Hence, these laboratories only perform a part of the diagnostic strategy outside office hours. This diagnostic strategy could lead to delayed treatment of malaria patients, especially if a less sensitive screening assay is used. Although ICTs are easy to perform, fast and relatively cheap, a major constraint of ICTs is false negative results. False negative results can occur in low parasitaemia as ICTs do not reliably detect densities below 100 parasites/µL in *P. falciparum* infections (the most life-threatening species), and *P. vivax* densities below 200 parasites/µL [13, 14]. Although the detection limit of ICTs for the other *Plasmodium* species that can infect humans are less clear, false negative ICT results for these *Plasmodium* species are likely to occur due to an often low parasitaemia. False negative results can also occur in high parasite densities for example in case of *P. falciparum* hyperparasitaemia, caused by the prozone effect due to an excess of either antigens or antibodies [15]. False negative results may also be caused by inaccurate interpretation [16]. Finally, genetic variations of *Plasmodium* species can lead to false negative ICT results, for example deletions or mutations within the HRP-2 gene of *P. falciparum* can lead to false negative results in *P. falciparum* histidine rich protein 2 (pfHRP-2) based ICTs [17, 18]. The LAMP malaria assay does not bear this problem and has a very high negative predictive value, but it is expensive and, compared to the ICT, a relatively time consuming method [19, 20]. A single laboratory uses the QBC assay as a screening assay as it provides fast, reliable results, but it is expensive and requires 24/7 availability of highly qualified technicians [20]. Therefore, the three screening assays used in the Dutch clinical laboratories, each have their own advantages and disadvantages.

The four guidelines for the diagnosis of malaria (NVP, BSH, CDC and WHO) compared in this study all consider microscopic examination of thick and thin blood films as the backbone of the laboratory diagnosis of malaria, which is reflected in the results of this study as microscopic examination of thick and thin blood films is part of the diagnostic strategy in all laboratories, apart from two laboratories that only perform a screening assay and subsequently send the material to an external laboratory for an unknown further examination. Referral to an external laboratory does result in delay of examination, but this delay may be acceptable as some laboratories are located in close proximity to each other in the densely populated areas of the Netherlands. The guidelines are not unanimous in when to perform microscopic examination of thick and thin blood films in combination with an ICT, as the NVP guideline advices to always examine the thick and thin blood film promptly in case of a positive ICT, however outside office hours, in case of a negative ICT microscopic examination of the thick and thin blood films may follow the next morning in the absence of a serious clinical suspicion of malaria [12]. Conversely, the BSH guideline, allows, in case an ICT is used outside office hours and “when there is a relatively inexperienced observer or when pressure of work out-of-hours prevents adequate microscopic assessment”, a positive ICT to be confirmed by microscopic examination the next morning [10]. Thereby the BSH guideline highlights the importance of confirmation of positive results of screening assays, which is recommended by all guidelines. Confirmation of positive screening assay results is particularly important if an ICT is used, as false positive ICT results have been reported. Hence, confirmation of positive screening assay results is essential to prevent false-positive results, which can lead to incorrect treatment. Furthermore, the CDC recommends that all ICTs are followed-up with microscopic examination of thick and thin blood films to confirm the results, but the degree of speed and a distinction between office hours and outside office hours is not mentioned in the consulted information [21] and the WHO recommends a prompt malaria diagnosis by either ICT in remote areas with limited access to good quality microscopy services’ or microscopic examinations of thick and thin blood films [22].

Substantial differences were also found between the surveyed laboratories in the method of preparing and examining thick and thin blood films, which is of interest as good quality thick and thin blood films are essential for proper subsequent microscopic analysis. Firstly, a substantial variation between the laboratories and between the guidelines was found in the used Giemsa stain concentration and the staining time of thick and thin blood films. A correlation between lower Giemsa concentrations and longer staining time was found. A slow staining method with a relatively low Giemsa concentration and long staining time yields better quality stained slides with less debris, and therefore, allows an easier detection and identification of *Plasmodium* species. The disadvantage, however, is that it takes more time, and thus may delay the malaria diagnosis. Depending on the level of experience of the observer and the use of other (rapid) diagnostic tests, laboratories can make a well-considered choice between speed of the staining method and the resulting quality of thick and thin blood films that will affect the sensitivity of the subsequent microscopic examination. Secondly, differences were found in the volume and diameter of blood used for the thick film. The BSH and CDC guideline do not recommend a certain volume of blood, moreover the BSH guideline does not give a recommendation about the diameter of the thick film either [10, 11]. The NVP and WHO agree on the volume of 6 µL blood and a diameter of 12 mm. However, they differ in the minimum number of microscopic fields, 200 versus 100 respectively, that should be examined before a thick film can be regarded as negative, which means that the theoretical limit of detection of the thick film differs by a factor of two [8, 9, 12]. Most surveyed laboratories use 6 µL of blood, as recommended by the NVP and WHO guidelines, however, the diameter of blood and number of fields examined differ between the laboratories. This shows that substantial differences between laboratories are present on how intensively the thick and thin blood films are examined, and therefore, the theoretical sensitivity of microscopic examination of the thick film differs by at least a factor 20 between clinical laboratories (1 to 22 parasites/µL). These differences in sensitivity of thick film examination are clinically relevant if thick film examination is the most sensitive test in the diagnostic strategy to detect malaria cases with a low parasitaemia that also occur in travelers in non-endemic countries [23]. These differences in the microscopic examination of thick and thin blood films will be further affected by the level of experience of the technician, but a reduced sensitivity and/or reliability can be compensated by combining microscopic thick and thin blood film examination with other methods with a high negative predictive value, such as LAMP, QBC or rtPCR. However, the guidelines are not clear on, or are lacking information about, how and when other methods such as LAMP, rtPCR and QBC can be implemented in the diagnostic strategy along with microscopic examination of thick and thin blood films. Thirdly, differences between laboratories and guidelines were found in the determination of parasitaemia, which will result in differences in the accuracy of the reported parasitaemia. The reason and clinical impact of the differences in the determination of the parasitaemia are unknown. The advantage of counting a large number of infected RBCs or parasites in thin and thick blood films, respectively, is that the accuracy of the parasitaemia increases. On the other, counting more infected RBCs or parasites will take more examination time which could result in delay in start of treatment. Therefore, it is important to optimize accuracy and speed of analysis and apparently distinct laboratories have chosen for different protocols.

The variations found in diagnostic strategies and microscopic examinations may be explained by differences in guidelines of the most important authorities. They could also be the result of a well-considered decision, based on different circumstances in the laboratories that perform malaria examinations in a clinical setting; for example the number of malaria requests and number of malaria positive patients per year and therefore the level of experience of the laboratory technicians, availability of trained technicians within and outside office hours, the financial resources of the laboratory and the maximum allowed costs of the entire diagnostic strategy. Table 3 shows an overview of the strengths and limitations of each diagnostic method and the possible function of each method in the diagnostic strategy. It is possible that the observed differences among laboratories in microscopic examination of thick and thin blood films and especially their diagnostic strategy are the result of carefully weighed decisions in order to optimize sensitivity, reliability and speed to these various circumstances. If this assumption is true, in these settings, more stringent standardization could unfavorably lead to the loss of accuracy and sensitivity of the laboratory diagnosis of malaria.

**Table 3:** Strengths and limitations compared to the gold standard (microscopic examination of thick and thin blood films) of additional methods to diagnose malaria

	Microscopic examination of thick and thin blood films	ICT	LAMP assay	QBC examination	rtPCR
Sensitivity (detection limit)*	Reference method	− −	+ + +	+ +	+ + +
*Plasmodium* species determination	Reference method	−	− − −	− −	+ + +
*P. falciparum determination*	Reference method	±	− − −	−	+ + +
Parasitaemia determination	Reference method	− −	− − −	− −	−
Speed of examination	Reference method	+ + +	±	+ + +	− −
Simplicity of examination	Reference method	+ + +	+ +	− −	+
Low cost	Reference method	+ + +	−	−	−
Possible function in diagnostic strategy	All-round method that can be used as stand-alone method if experienced technicians are available	Fast and simple screening assay that can be organized close to the patient	Reliable exclusion of malaria by less experienced technicians	Fast screening for malaria parasites if experienced technicians are available	Slow confirmation assay in case other methods are non-conclusive

ICT: immunochromatographic test; LAMP: loop mediated isothermal amplification; QBC: Quantitative Buffy Coat; rtPCR: real-time polymerase chain reaction

*Based on an experienced laboratory technician; +, ± , −: better, equal or worse compared to the reference method microscopic examination of thick and thin blood films, respectively

**Fig. 4:**
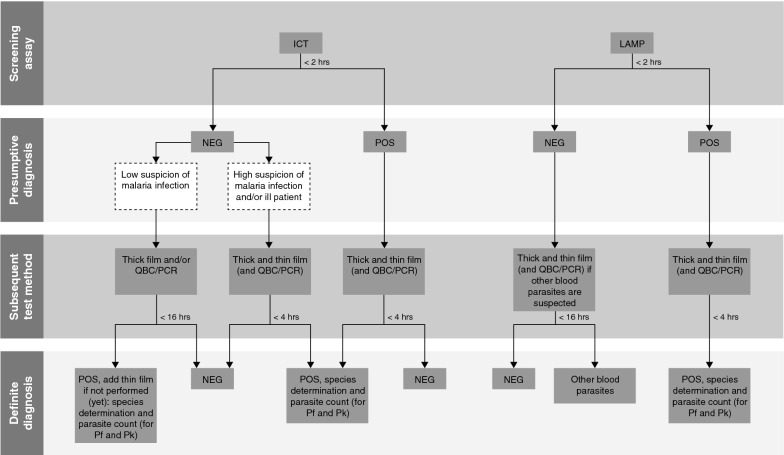
Algorithm with requirements for the laboratory diagnosis of malaria. The given time limits (for example ‘< 2 h’) indicate the maximum amount of time between submission of blood samples and test result. hrs: hours; POS: positive; NEG: negative; ICT: immunochromatographic test; LAMP: loop-mediated isothermal amplification; QBC: Quantitative Buffy Coat; Pf: *Plasmodium falciparum*; Pk: *Plasmodium knowlesi*

Nevertheless, some degree of standardization on the laboratory diagnosis of malaria can be composed, taking into account various circumstances as described above, to ensure fast and accurate test results. Figure 4 displays a recommended algorithm with requirements for speed and quality of a presumptive and definite diagnosis of malaria in symptomatic patients in malaria non-endemic, high resource settings: (I) use of an easy to use screening assay close to the patient to provide a presumptive diagnosis 24/7 as quickly as possible (e.g. within two hours after submitting blood samples). Providing a fast presumptive diagnosis is important to ensure immediate treatment of severe malaria cases. The screening assay must be very easy to use such as an ICT, LAMP or better methods that become available in the future and do not require highly skilled technicians, such as fluorescence flow cytometry [24]. The speed requirement and type of subsequent test methods depend on the outcome of the screening assay and the sensitivity and specificity of the screening assay used. (II) at all times a positive screening assay result must directly (e.g. within 4 h after submitting blood samples) be followed by microscopic examination of thick and thin blood films to determine the *Plasmodium* species and, in case of *P. falciparum* and/or *P. knowlesi*, the parasitaemia to ensure that the correct definitive treatment is given; (III) Although an ICT is a sensitive method, with a theoretical detection limit of 100 parasites/µL [13], a negative ICT result should be confirmed with another test method to rule out false negative results [15–18]. In case a patient is seriously ill and clinically highly suspected of a malaria infection, a false negative ICT result must be ruled out directly (e.g. within 4 h after submitting blood samples) by microscopic examination of the thick and thin blood films. In case a patient is not clinically highly suspected of a malaria infection, the subsequent test method with a limit of detection below 10 parasites/µL (e.g. thick film, QBC or rtPCR) can be performed later (e.g. outside office hours the subsequent test can be performed the next morning); (IV) In case LAMP is used as screening assay, a negative result does not have to be confirmed at all times by another test method, due to the very high negative predictive value of the LAMP. Only in case of a suspected infection with other blood parasites, such as filarial worms or *Trypanosoma* species subsequent microscopic examinations are recommended (either directly or in case of outside office hours it can be performed the next morning). Finally, for patients with a persisting, strong suspicion for malaria despite a negative test result, it is advised to repeat testing as errors can never completely be excluded by any strategy.

Furthermore the method of preparing and examining thick and thin blood films should be determined by the aim of microscopic examination of blood films. If the thick film is the most sensitive element in the diagnostic strategy used, it is recommended to perform a high quality staining method such as the ‘slow’ Giemsa staining method with 2.5–4% Giemsa concentration and 45–60 min of staining. A sufficient number of microscopic fields must be examined to reach a theoretical detection limit of 5 parasites/µL. If the thick film is used for other purposes, such as the determination of parasitaemia, a quick staining method can be used such as DiffQuik® or the ‘fast’ Giemsa staining method with 10% Giemsa concentration and 8–40 min of staining. In this case, a smaller number of microscopic fields can be examined.

### Limitations

Overall, the response rate to the survey was high (67/77, 87.0%), but the response rate to one component of the survey, the volume of blood used for and the diameter of the thick film, however was low (21/77, 27.3%). Possibly this could have resulted in a participation bias. For example, it is probable that laboratories who standardized this specific method responded more often to the questions, and therefore, represent less generalizable results. As the responses were self-reported, and not verified by the researchers, questions could have been misunderstood, affecting the way responses are provided and resulting in a potential risk of a response bias.

A non-significant trend was found between the number of malaria requests per technician per laboratory per year and the overall performance score in the malaria EQAS. However, the examination of EQA samples may not reflect the examination of real patient samples, because it cannot be fully excluded that in case of an EQAS sample the blood films are examined by multiple, well trained technicians and staff members that make a joint judgement, versus the patient samples that are often examined by one technician only.

## Conclusions

Altogether it can be concluded that, although microscopic examination of thick and thin films is the backbone of the laboratory diagnosis of malaria in symptomatic patients in a high-resource country and non-endemic setting, substantial differences exist between clinical laboratories in the method of preparing and examining of thick and thin blood films and which type of methods are performed and when, especially outside office hours in case of a negative test result of the screening assay. The observed variations may be explained by differences in the four guidelines of the most important authorities, but could also be the result of a well-considered decision based on the different circumstances in the laboratories that perform diagnostic examinations for malaria in symptomatic patients. The impact of these variations on the quality of the entire diagnostic strategy of malaria is unknown and difficult to assess due to the tremendous amount of variations in strategies. Therefore, each laboratory has to be critical on their diagnostic strategy, taking into account the strengths and limitations of their facilities and the experience of their laboratory technicians with malaria in order to warrant reliable results. In addition, guidelines could give more guidance to clinical laboratories on the microscopic examination of thick and thin blood films and what (combined) diagnostic methods can be performed and when. These guidelines should especially take into account the existing differences in the level of experience of laboratory technicians and the availability of technicians within and outside office hours, to ensure optimal and reliable laboratory diagnosis of malaria in diverse circumstances.

## Supplementary Information

**Additional file 1.** Questionnaires.

**Additional file 2.** Theoretical sensitivity of thick and thin blood films of participating laboratories (n = 50, MD = 27). missing data;. = no calculation possible due to missing data; ND = not determined for the detection of parasites; NA = not applicable; * the size of 1 euro coin; ** the size of a dime; ^ 1000× magnification and FN 18 of the ocular is assumed, based on the use of a 100× objective.

**Additional file 3.** Determination of parasitaemia. A shows the amount of WBCs counted in the thick films by the surveyed laboratories for the determination of parasitaemia. The guidelines count the following amount of WBCs: NVP (200 WBCs or 500 WBCs if < 10 parasites are counted); BSH (MD); CDC 1000 WBCs or 500 parasites, whichever comes first); WHO (200 WBCs or 500 WBCs if < 100 parasites are counted). B shows the amount of RBCs counted in the thin films by the laboratories for the determination of parasitaemia. The guidelines count the following amount of RBCs: NVP (10,000 RBCs); BSH (1000 RBCs); CDC (500 RBCs, 2000 if parasitaemia is < 1%); WHO (5000 RBCs). Abbreviations; MD = missing data; NP = not performed; WBCs = white blood cells; RBCs = red blood cells.

## Data Availability

All data generated or analyzed during this study are included in this published article and its supplementary information files.

## Abbreviations

BSH: British Society of Haematology
CDC: Center for Disease Control and Prevention
EQAS: External quality assessment scheme
FN: Field Number
HRP-2: Histidine-rich protein 2
ICT: Immunochromatographic test
LAMP: Loop-mediated isothermal amplification
Mm: Millimeter
MD: Missing data
NVP: Dutch Society for Parasitology
QBC: Quantitative Buffy Coat
RBCs: Red blood cells
rtPCR: Real-time polymerase chain reaction
SKML: Dutch Foundation for Quality Assessment in Medical Laboratories
WBCs: White blood cells
WHO: World Health Organization
µL: Microlitre
