# Supplementary material for: Malaria diagnosis in a malaria non-endemic high-resource country: high variation of diagnostic strategy in clinical laboratories in the Netherlands
Source: Malar J. 2021 Oct 19;20:411. doi: 10.1186/s12936-021-03889-7 (PMC8524939; doi:10.1186/s12936-021-03889-7)
Supplement: Supplementary file 1 — Additional file 1. Questionnaires. [file 12936_2021_3889_MOESM1_ESM.docx]

*[Dutch questionnaire no. 1; March 2018]*

De SKML-sectie parasitologie is benieuwd naar de in Nederland gebruikte methoden voor het kleuren van bloeduitstrijkjes, omdat hiervoor verschillende methoden bestaan. De verdiepingsvragen van deze rondzending zijn daarom gericht op de in uw laboratorium gebruikte kleurmethode voor bloeduitstrijkjes.

*Vragen gebruikt voor analyse*

**Vraag 1**

Welke kleurstof gebruikt uw laboratorium voor het kleuren van bloeduitstrijkjes?

1. Giemsa
2. Diff Quick
3. Diff Quick + bij twijfel/problemen in combinatie met Giemsa
4. Anders (licht toe bij opmerkingen)

**Vraag 2**

In welke concentratie gebruikt uw laboratorium de kleurstof? Graag beantwoorden met getal in volumeprocenten (bij gebruik Giemsa 1:25 is dat dus 4% v/v).

**Vraag 5**

Hoelang worden bloeduitstrijkjes in uw laboratorium gekleurd (graag antwoorden met een getal in minuten; half uur dus graag rapporteren als 30)?

*Vragen niet gebruikt voor analyse (niet eenduidig geformuleerd of geen interessante bevindingen)*

**Vraag 3**

Wat is de samenstelling van de buffer waarin de kleurstof wordt verdund. Graag de componenten in chemische formule beschrijven met de concentratie in millimolair (mM). (Fysiologisch zout graag rapporteren als NaCl 150mM).

**Vraag 4**

Wat is de pH van de door uw laboratorium gebruikte buffer (indien de oplossing waarin de kleurstof wordt verdund niet gebufferd is en niet op pH wordt gesteld, graag 0 als antwoord rapporteren)?

**Vraag 6**

Voegt uw laboratorium nog specifieke stoffen toe aan de kleuroplossing voor bloeduitstrijkjes (zoals bijvoorbeeld detergentia zoals Trition X-100)? Zo ja, graag de toevoegde stoffen en hun concentratie in de kleuroplossing rapporteren.

*[Dutch to English translated questionnaire no. 1; March 2018]*

The SKML-section of parasitology is curious about which staining methods for thick and thin blood films are used in the Netherlands, because there are different methods for this. The in-depth questions of this EQAS distribution round therefore focus on the staining method for thick and thin blood films used in your laboratory.

*Questions used for analysis*

**Question 1**

What dye does your laboratory use for staining thick and thin blood films?

1. Giemsa
2. Diff Quick
3. Diff Quick + only if the Diff Quick stained films result in equivocal results in combination with Giemsa
4. Other (explain in comments)

**Question 2**

What concentration of the dye is used in your laboratory? Please answer with number in volume percentages (using Giemsa 1:25 equals 4% v / v).

**Question 5**

For how long are thick and thin blood films stained in your laboratory (please answer with a number in minutes; so please report a half hour as 30)?

*Questions not used for analysis (not clearly formulated or no interesting findings)*

**Question 3**

What is the composition of the buffer in which the dye is diluted. Please describe the components in chemical form with the concentration in millimolar (mM). (Please report physiological salt as NaCl 150mM).

**Question 4**

What is the pH of the buffer used by your laboratory (if the solution in which the dye is diluted is not buffered and not adjusted to pH, please report 0 in answer)?

**Question 6**

Does your laboratory add specific substances to the staining solution for thick and thin blood films (such as detergents such as Trition X-100)? If so, please report the additives and their concentration in the staining solution.

*[Dutch questionnaire no. 2; June 2019]*

**Vraag 1**

Hoeveel malaria onderzoeken worden er ongeveer PER JAAR uitgevoerd in uw laboratorium?

- <10
- 10-50
- 50-100
- 100-250
- 250-500
- >500

**Vraag 2**

Hoeveel patiënten worden er ongeveer PER JAAR met malaria gediagnosticeerd in uw laboratorium?

- <1
- 1-5
- 50-10
- 10-25
- 25-50
- >50

**Vraag 3**

Welke bepalingen worden in uw laboratorium DIRECT uitgevoerd bij alle aanvragen BINNEN kantoortijd? (mogelijk meerdere opties aan te vinken)

- - Detectie antigeen (ICT)
  - Dikke druppel
  - Uitstrijk
  - Quantitative Buffy Coat (QBC)
  - Real time PCR (commercieel of in-house)
  - LAMP test (merk zoals Meridian)

**Vraag 4**

Welke bepalingen worden in uw laboratorium DIRECT uitgevoerd bij alle aanvragen BUITEN kantoortijd? (mogelijk meerdere opties aan te vinken)

- - Detectie antigeen (ICT)
  - Dikke druppel
  - Uitstrijk
  - Quantitative Buffy Coat (QBC)
  - Real time PCR (commercieel of in-house)
  - LAMP test (merk zoals Meridian)

**Vraag 5**

Indien er BUITEN kantoortijd een NEGATIEF resultaat met de screeningsassay (bijvoorbeeld detectie antigeen of LAMP test) wordt gevonden, wat zijn dan de vervolgstappen de eerstvolgende dag? (mogelijk meerdere opties aan te vinken)

- - Geen vervolgonderzoek
  - Dikke druppel
  - Uitstrijk
  - Real time PCR (commercieel of in-house)
  - Quantitative Buffy Coat (QBC)
  - N.v.t. omdat de bepalingen binnen en buiten kantoortijd identiek zijn

**Vraag 6**

Indien er BUITEN kantoortijd een POSITIEF resultaat met de screeningsassay (bijvoorbeeld detectie antigeen of LAMP test) wordt gevonden, wat zijn dan de DIRECTE vervolg stappen? (meerdere opties aan te vinken)

- - Geen vervolgonderzoek
  - Dikke druppel
  - Uitstrijk
  - Real time PCR (commercieel of in-house)
  - Quantitative Buffy Coat (QBC)
  - Positieve bevindingen in de sneltest of LAMP test worden elders geconfirmeerd
  - N.v.t. omdat de bepalingen binnen en buiten kantoortijd identiek zijn
  - Anders, namelijk……..

**Vraag 7**

Is het maken van de dikke druppel gestandaardiseerd?

- 1. Nee
  2. Ja, vermeld bij opmerkingen het volume opgebracht bloed in uL en de diameter van de dikke druppel in mm.

**Vraag 8**

Dient er bij malaria onderzoek in uw laboratorium een bepaalde tijd of een bepaald aantal gezichtsvelden bekeken te worden voordat een NEGATIEVE uitslag gerapporteerd mag worden? Met andere woorden, is de microscopische beoordeling gestandaardiseerd?

1. Nee
2. Ja, gestandaardiseerd in tijd
3. Ja, gestandaardiseerd in aantal gezichtsvelden

**Vraag 9**

Wat is de kijktijd (min) óf aantal gezichtsvelden per DIKKE DRUPPEL in uw laboratorium?

- <5 min
- 5-10 min
- 10-15 min
- 15-20 min
- 20-25 min
- 25-30 min
- >30 min
- <50 gezichtsvelden
- 50-100 gezichtsvelden
- 100-150 gezichtsvelden
- 150-200 gezichtsvelden
- 200-250 gezichtsvelden
- 250-300 gezichtsvelden
- >300 gezichtsvelden

**Vraag 10**

Wat is de kijktijd (minuten) óf aantal gezichtsvelden per UITSTRIJK in uw laboratorium?

- <5 min
- 5-10 min
- 10-15 min
- 15-20 min
- 20-25 min
- 25-30 min
- >30 min
- <50 gezichtsvelden
- 50-100 gezichtsvelden
- 100-150 gezichtsvelden
- 150-200 gezichtsvelden
- 200-250 gezichtsvelden
- 250-300 gezichtsvelden
- >300 gezichtsvelden

**Vraag 11**

Met welke totale vergroting (objectief x oculair) worden de preparaten bekeken in uw laboratorium?

1. 500
2. 625
3. 1000
4. 1250
5. Anders, namelijk ……………

**Vraag 12**

Hoe wordt de parasitemie bij *P. falciparum* en *P. knowlesi* bepaald in het DIKKE DRUPPEL preparaat in uw laboratorium?

1. Dmv het aantal trofozoieten per aantal leukocyten (vermeld bij opmerkingen het aantal leukocyten dat minimaal geteld moet worden)
2. Anders, namelijk ………………
3. Niet van toepassing

**Vraag 13**

Hoe wordt de parasitemie bij *P. falciparum* en *P. knowlesi* bepaald in de UITSTRIJK?

1. Dmv het aantal trofozoieten per erytrocyten (vermeld bij opmerkingen het aantal erytrocyten dat geteld moet worden)
2. Anders, namelijk ………………
3. Niet van toepassing

*[Dutch to English translated questionnaire no. 2; June 2019]*

**Question 1**

How many malaria tests are performed in your laboratory approximately PER YEAR?

- <10
- 10-50
- 50-100
- 100-250
- 250-500
- >500

**Question 2**

How many patients are diagnosed with malaria in your laboratory approximately PER YEAR?

- <1
- 1-5
- 50-10
- 10-25
- 25-50
- >50

**Question 3**

Which examinations are carried out IMMEDIATELY in your laboratory for all requests WITHIN office hours? (it is possible to tick several options)

- - Antigen detection test (ICT)
  - Thick film
  - Thin film
  - Quantitative Buffy Coat (QBC)
  - Real time PCR (commercial or in-house)
  - LAMP test (brand like Meridian)

**Question 4**

Which examinations are carried out IMMEDIATELY in your laboratory for all requests OUTSIDE office hours? (it is possible to tick several options)

- - Antigen detection test (ICT)
  - Thick film
  - Thin film
  - Quantitative Buffy Coat (QBC)
  - Real time PCR (commercial or in-house)
  - LAMP test (brand like Meridian)

**Question 5**

If a NEGATIVE result with the screening assay (for example antigen detection test or LAMP test) is found OUTSIDE office hours, what are the next steps the next day? (it is possible to tick several options)

- - No follow-up examinations
  - Thick film
  - Thin film
  - Real time PCR (commercial or in-house)
  - Quantitative Buffy Coat (QBC)
  - Not applicable because the examinations within and outside office hours are identical

**Question 6**

If a POSITIVE result with the screening assay (e.g. antigen detection test or LAMP test) is found OUTSIDE office hours, what are the IMMEDIATE follow-up steps? (it is possible to tick several options)

- - No follow-up examinations
  - Thick film
  - Thin film
  - Real time PCR (commercial or in-house)
  - Quantitative Buffy Coat (QBC)
  - Positive test results of the ICT or LAMP test are confirmed elsewhere
  - Not applicable because the examinations within and outside office hours are identical
  - Other, namely……..

**Question 7**

Is the preparation of the thick film standardized?

1. No
2. Yes, in the comments, state the volume of blood applied in µL and the diameter of the thick film in mm.

**Question 8**

In your laboratory, does the examination of the films require a certain time or number of microscopic fields to be viewed before a NEGATIVE result can be reported? In other words, is the microscopic assessment standardized?

1. No
2. Yes, standardized in time
3. Yes, standardized in number of microscopic fields

**Question 9**

What is the duration of examination (min) ór number of microscopic fields per THICK FILM in your laboratory?

- <5 min
- 5-10 min
- 10-15 min
- 15-20 min
- 20-25 min
- 25-30 min
- >30 min
- <50 microscopic fields
- 50-100 microscopic fields
- 100-150 microscopic fields
- 150-200 microscopic fields
- 200-250 microscopic fields
- 250-300 microscopic fields
- >300 microscopic fields

**Question 10**

What is the duration of examination (min) ór number of microscopic fields per THIN FILM in your laboratory?

- <5 min
- 5-10 min
- 10-15 min
- 15-20 min
- 20-25 min
- 25-30 min
- >30 min
- <50 microscopic fields
- 50-100 microscopic fields
- 100-150 microscopic fields
- 150-200 microscopic fields
- 200-250 microscopic fields
- 250-300 microscopic fields
- >300 microscopic fields

**Question 11**

With what total magnification (objective x eyepiece) are the specimens viewed in your laboratory?

1. 500
2. 625
3. 1000
4. 1250
5. Other, namely ……………

**Question 12**

How is the parasitaemia of *P. falciparum* and *P. knowlesi* determined in the THICK FILM in your laboratory?

1. By the number of trophozoites per number of leukocytes (state the minimum number of leukocytes that must be counted in the comments)
2. Other, namely ………………
3. Not applicable

**Question 13**

How is the parasitaemia of *P. falciparum* and *P. knowlesi* determined in the THIN FILM in your laboratory?

1. By the number of trophozoites per number of erythrocytes (indicate the number of erythrocytes to be counted in comments)
2. Other, namely ………………
3. Not applicable

*[Dutch questionnaire no. 3; September 2019]*

**Vraag 1**Onderzoek naar malaria antigeen d.m.v. malaria antigeentest?

1. Nee
2. Ja, type/merk ……..

*[Dutch to English translated questionnaire no. 3; September 2019]*

**Question 1**Malaria antigen detection by means of a malaria antigen test?

1. No
2. Yes, type/brand ……..
