# Supplementary material for: Malaria diagnosis in a malaria non-endemic high-resource country: high variation of diagnostic strategy in clinical laboratories in the Netherlands
Source: Malar J. 2021 Oct 19;20:411. doi: 10.1186/s12936-021-03889-7 (PMC8524939; doi:10.1186/s12936-021-03889-7)
Supplement: Supplementary file 2 — Additional file 2. Theoretical sensitivity of thick and thin blood films of participating laboratories (n = 50, MD = 27). missing data;. = no calculation possible due to missing data; ND = not determined for the detection of parasites; NA = not applicable; * the size of 1 euro coin; ** the size of a dime; ^ 1000× magnification and FN 18 of the ocular is assumed, based on the use of a 100× objective. [file 12936_2021_3889_MOESM2_ESM.docx]

|  |  |  | *Thick film* | | | | | | | |  | *Thin film* | | | | |
| --- | --- | --- | --- | --- | --- | --- | --- | --- | --- | --- | --- | --- | --- | --- | --- | --- |
|  |  |  |  |  |  |  |  |  |  |  |  |  |  |  |  |  |
| Guideline/ laboratory | Total magnification (ocular x objective) | Theoretical field number ocular | Volume of thick film | Diameter of thick film | Theoretical thickness | Surface of one field (with magnification) | Number of fields examined | Theoretical total examined surface | Theoretical total examined volume | Theoretical limit of detection (based on 2 trophozoites) |  | Theoretical fields examined | Theoretical RBCs viewed | Theoretical total examined volume | Theoretical limit of detection (based on 2 trophozoites) | Theoretical part of volume examined compared to thick film |
| **Guideline** | *** | *mm* | *µL* | *mm* | *µL/mm^2^* | mm^2^ | *number* | *mm^2^* | *µL* | trophozoites /µL |  | *number* | *number* | *µL* | trophozoites /µL | *%* |
| *NVP* | 1,000 | 18^ | 6 | 12 | 0.05 | 0.03 | 200 | 5.1 | 0.3 | 7.41 |  | ND | . | . | . | . |
| *BSH* | 1,000^ | 18^ | several drops | - | . | . | 200 | . | . | . |  | ND | . | . | . | . |
| *CDC* | 1,000^ | 18^ | small drop | 17.9** | . | . | 100 - 300 | . | . | . |  | - | . | . | . | . |
| *WHO* | 1,000 | 18^ | 6 | 12 | 0.05 | 0.03 | 100 | 2.5 | 0.1 | 14.29 |  | ND | . | . | . | . |
| **Range** |  |  |  |  |  |  |  |  |  |  |  |  |  |  |  |  |
| *Minimum* | 500 | 16 | 6 | 6 | 0.02 | 0.02 | 75 | 2.9 | 0.09 | 1.2 |  | 40 | 10,000 | 0.002 | 1000.0 | 0.8% |
| *Maximum* | 1,250 | 18 | 15 | 23.25* | 0.21 | 0.10 | 413 | 34.4 | 1.65 | 21.9 |  | 600 | 281,500 | 0.056 | 35.5 | 10.3% |
| *Mean* | NA | NA | 8 | 14 | 0.06 | 0.04 | 207 | 8 | 0.47 | 7.4 |  | 190 | 52,048 | 0.010 | 192.1 | 3.4% |
| *Median* | NA | NA | 6 | 13 | 0.05 | 0.03 | 200 | 5 | 0.31 | 6.5 |  | 175 | 45,000 | 0.009 | 222.2 | 2.9% |
| **Laboratory** |  |  |  |  |  |  |  |  |  |  |  |  |  |  |  |  |
| A | 1,000 | 18 | 6 | 20 | 0.02 | 0.03 | 188 | 4.8 | 0.09 | 21.9 |  | 188 | 46,875 | 0.009 | 213.3 | 10.3% |
| B | 1,000 | 18 | - | - | . | . | . | . | . | . |  | 263 | 65,625 | 0.013 | 152.4 | . |
| C | 500 | 18 | 6 | 15 | 0.03 | 0.10 | 175 | 17.8 | 0.60 | 3.3 |  | 175 | 87,500 | 0.018 | 114.3 | 2.9% |
| D | 1,000 | 18 | 6 | 6 | 0.21 | 0.03 | 200 | 5.1 | 1.08 | 1.9 |  | ND | . | . | . | . |
| E | 1,000 | 18 | - | - | . | . | . | . | . | . |  | 338 | 84,375 | 0.017 | 118.5 | . |
| F | 625 | 16 | - | - | . | . | . | . | . | . |  | 113 | 45,000 | 0.009 | 222.2 | . |
| G | 500 | 18 | - | - | . | . | . | . | . | . |  | 188 | 93,750 | 0.019 | 106.7 | . |
| H | 1,000 | 18 | - | - | . | . | . | . | . | . |  | 188 | 46,875 | 0.009 | 213.3 | . |
| I | 1,000 | 18 | 6 | 13 | 0.05 | 0.03 | 225 | 5.7 | 0.26 | 7.7 |  | ND | . | . | . | . |
| J | 1,000 | 18 | - | 10 | . | . | . | . | . | . |  | 75 | 18,750 | 0.004 | 533.3 | . |
| K | 1,000 | 18 | - | - | . | . | . | . | . | . |  | 275 | 68,750 | 0.014 | 145.5 | . |
| L | 1,250 | 16 | 6 | 13 | 0.05 | 0.02 | 338 | 6.8 | 0.31 | 6.5 |  | 74 | 14,800 | 0.003 | 675.7 | 1.0% |
| M | 1,000 | 18 | 6 | 15 | 0.03 | 0.03 | 200 | 5.1 | 0.17 | 11.6 |  | 200 | 50,000 | 0.010 | 200.0 | 5.8% |
| N | 1,000 | 18 | 6 | 13 | 0.05 | 0.03 | 188 | 4.8 | 0.22 | 9.3 |  | ND | . | . | . | . |
| O | 1,000 | 18 | - | - | . | . | . | . | . | . |  | 263 | 65,625 | 0.013 | 152.4 | . |
| P | 500 | 18 | 6 | 15 | 0.03 | 0.10 | 338 | 34.4 | 1.17 | 1.7 |  | 563 | 281,500 | 0.056 | 35.5 | 4.8% |
| Q | 500 | 18 | 6 | 13 | 0.05 | 0.10 | 225 | 22.9 | 1.04 | 1.9 |  | - | . | . | . | . |
| R | 500 | 18 | - | - | . | . | . | . | . | . |  | 125 | 31,250 | 0.006 | 320.0 | . |
| S | 625 | 16 | - | - | . | . | . | . | . | . |  | 413 | 45,000 | 0.009 | 222.2 | . |
| T | 1,000 | 18 | 6 | 12 | 0.05 | 0.03 | 200 | 5.1 | 0.27 | 7.4 |  | ND | . | . | . | . |
| U | 1,000 | 18 | - | - | . | . | . | . | . | . |  | 40 | 10,000 | 0.002 | 1000.0 | . |
| V | 1,000 | 18 | 6 | 11 | 0.06 | 0.03 | 200 | 5.1 | 0.32 | 6.2 |  | ND | . | . | . | . |
| W | 1,000 | 18 | 10 | 23.25 | 0.02 | 0.03 | 200 | 5.1 | 0.12 | 16.7 |  | 75 | 18,750 | 0.004 | 533.3 | 3.1% |
| X | 1,000 | 18 | 6 | 12 | 0.05 | 0.03 | 200 | 5.1 | 0.27 | 7.4 |  | ND | . | . | . | . |
| Y | 1,000 | 18 | - | - | . | . | . | . | . | . |  | 113 | 28,125 | 0.006 | 355.6 | . |
| Z | 1,000 | 18 | 10 | 9 | 0.16 | 0.03 | 413 | 10.5 | 1.65 | 1.2 |  | 413 | 103,125 | 0.021 | 97.0 | 1.3% |
| AA | 1,000 | 18 | - | - | . | . | . | . | . | . |  | 75 | 18,750 | 0.004 | 533.3 | . |
| AB | 1,000 | 18 | 6 | - | . | . | . | . | . | . |  | 49 | 12,250 | 0.002 | 816.3 | . |
| AC | 1,000 | 18 | - | 20 | . | . | . | . | . | . |  | 263 | 65,625 | 0.013 | 152.4 | . |
| AD | 1,000 | 18 | - | - | . | . | . | . | . | . |  | 113 | 28,125 | 0.006 | 355.6 | . |
| AE | 1,000 | 18 | - | - | . | . | . | . | . | . |  | 263 | 65,625 | 0.013 | 152.4 | . |
| AF | 1,000 | 18 | 6 | 12 | 0.05 | 0.03 | 200 | 5.1 | 0.27 | 7.4 |  | - | . | . | . | . |
| AG | 1,000 | 18 | - | 15 | . | . | . | . | . | . |  | 49 | 12,250 | 0.002 | 816.3 | . |
| AH | 1,000 | 18 | - | - | . | . | . | . | . | . |  | 263 | 65,625 | 0.013 | 152.4 | . |
| AI | 1,000 | 18 | 15 | 14 | 0.10 | 0.03 | 135 | 3.4 | 0.33 | 6.0 |  | 135 | 33,750 | 0.007 | 296.3 | 2.0% |
| AJ | 1,000 | 18 | - | - | . | . | . | . | . | . |  | 338 | 84,375 | 0.017 | 118.5 | . |
| AK | 1,000 | 18 | - | - | . | . | . | . | . | . |  | 175 | 43,750 | 0.009 | 228.6 | . |
| AL | 1,000 | 18 | - | - | . | . | . | . | . | . |  | 74 | 18,500 | 0.004 | 540.5 | . |
| AM | 1,000 | 18 | - | - | . | . | . | . | . | . |  | 200 | 50,000 | 0.010 | 200.0 | . |
| AN | 1,000 | 18 | 6 | 12 | 0.05 | 0.03 | 113 | 2.9 | 0.15 | 13.2 |  | ND | . | . | . | . |
| AO | 1,000 | 18 | 6 | 10 | 0.08 | 0.03 | 200 | 5.1 | 0.39 | 5.1 |  | 200 | 50,000 | 0.010 | 200.0 | 2.6% |
| AP | 1,000 | 18 | 6 | - | . | . | . | . | . | . |  | 75 | 18,750 | 0.004 | 533.3 | . |
| AQ | 1,000 | 18 | 6 | - | . | . | . | . | . | . |  | 600 | 150,000 | 0.030 | 66.7 | . |
| AR | 1,000 | 18 | - | - | . | . | . | . | . | . |  | 75 | 18,750 | 0.004 | 533.3 | . |
| AS | 625 | 16 | 15 | 16 | 0.07 | 0.08 | 75 | 6.0 | 0.45 | 4.4 |  | ND | . | . | . | . |
| AT | 1,000 | 18 | - | - | . | . | . | . | . | . |  | 188 | 46,875 | 0.009 | 213.3 | . |
| AU | 1,000 | 18 | 15 | - | . | . | . | . | . | . |  | 74 | 18,500 | 0.004 | 540.5 | . |
| AV | 1,000 | 18 | 6 | 10 | 0.08 | 0.03 | 225 | 5.7 | 0.44 | 4.6 |  | 74 | 18,500 | 0.004 | 540.5 | 0.8% |
| AW | 1,000 | 18 | 12 | 15 | 0.07 | 0.03 | 113 | 2.9 | 0.19 | 10.3 |  | 113 | 28,125 | 0.006 | 355.6 | 2.9% |
| AX | 1,000 | 18 | - | - | . | . | . | . | . | . |  | 113 | 28,125 | 0.006 | 355.6 | . |
