# Supplementary material for: Malaria diagnosis in a malaria non-endemic high-resource country: high variation of diagnostic strategy in clinical laboratories in the Netherlands
Source: Malar J. 2021 Oct 19;20:411. doi: 10.1186/s12936-021-03889-7 (PMC8524939; doi:10.1186/s12936-021-03889-7)
Supplement: Supplementary file 3 — Additional file 3. Determination of parasitaemia. A shows the amount of WBCs counted in the thick films by the surveyed laboratories for the determination of parasitaemia. The guidelines count the following amount of WBCs: NVP (200 WBCs or 500 WBCs if < 10 parasites are counted); BSH (MD); CDC 1000 WBCs or 500 parasites, whichever comes first); WHO (200 WBCs or 500 WBCs if < 100 parasites are counted). B shows the amount of RBCs counted in the thin films by the laboratories for the determination of parasitaemia. The guidelines count the following amount of RBCs: NVP (10,000 RBCs); BSH (1000 RBCs); CDC (500 RBCs, 2000 if parasitaemia is < 1%); WHO (5000 RBCs). Abbreviations; MD = missing data; NP = not performed; WBCs = white blood cells; RBCs = red blood cells. [file 12936_2021_3889_MOESM3_ESM.docx]

| **A. Determination of parasitaemia in thick film,  based on the number of WBCs** | | **B. Determination of parasitaemia in thin film,  based on the number of RBCs** | |
| --- | --- | --- | --- |
| *Category* | *Number of laboratories* | *Category* | *Number of laboratories* |
| 100 | 2 | 100 - <1,000 | 1 |
| 200 | 8 | 1,000 - <5,000 | 8 |
| 200;500 (if < 10) | 14 | 5,000 - <10,000 | 5 |
| 500 | 1 | 10,000 | 25 |
| 1,000 | 0 | >10,000 | 0 |
| NP | 14 | MD | 38 |
| MD | 38 |  |  |
| *Range* |  | *Range* |  |
| minimum | 100 | minimum | 100 |
| maximum | 500 | maximum | 10,000 |
| Mean | NA | Mean | 7,656 |
| Median | NA | Median | 10,000 |
| *Number of WBCs* | *Guidelines* | *Number of RBCs* | *Guidelines* |
| 200;500 (if <10 parasites) | NVP | 10,000 | NVP |
| MD | BSH | 1,000 | BSH |
| 1,000 | CDC | 500;2,000 (if parasitaemia < 1%) | CDC |
| 200;500 (if <100 parasites) | WHO | 5,000 | WHO |
| TOTAL | 77 | TOTAL | 77 |
